# MUC1 promotes RIF by regulating macrophage ROS-SHP2 signaling pathway to up-regulate inflammatory response and inhibit angiogenesis

**DOI:** 10.18632/aging.205560

**Published:** 2024-02-26

**Authors:** Rongna Liu, Lin Chen, Xin Zhao, Lili Bao, Ruixia Wei, Xiaohua Wu

**Affiliations:** 1 Department of Obstetrics and Gynecology, Hebei Medical University, Shijiazhuang 050017, China; 2 Department of Obstetrics and Gynecology, Bethune International Peace Hospital, Shijiazhuang 050082, Hebei, China; 3 Department of Reproductive Medicine Centre, Shijiazhuang Obstetrics and Gynecology Hospital, Shijiazhuang 050017, China

**Keywords:** recurrent implantation failure, MUC1, ROS-SHP2 signaling pathway, inflammatory response

## Abstract

Objective: To explore the effect of MUC1 on recurrent implantation failure (RIF) and its molecular mechanism.

Methods: Bioinformation analysis was used to find possible molecular mechanisms of specific genes in the pathogenesis of RIF. The number of M1 and M2 macrophages was measured by flow cytometry. Immunohistochemical staining and western blotting were used to detect the expression of related proteins. Angiogenesis capacity was measured by cell tube-formation assay.

Results: Bioinformatics analysis results suggest that MUC1 may play an important role in RIF. The results of flow cytometry showed that compared with NC group, M1 macrophages increased significantly and M2 macrophages decreased significantly in MUC1 OE group. The results of immunohistochemical staining showed that MUC1 could inhibit the expression of VEGF. Western blotting results showed that MUC1 could significantly increase the expression of P22, P47, gp91, p-TBK1, IFNγ and IL-1β, and decrease the expression of p-SHP2, p-PI3K, p-mTOR, HIF1α and VEGF. After the addition of ROS inhibitor and PI3K inhibitor, the effect of MUC1 on the above proteins was eliminated. The results of tube formation experiments showed that MUC1 could inhibit vascular formation.

Conclusion: As a promising biomarker for the diagnosis of RIF, MUC1 can promote RIF by regulating macrophage ROS-SHP2 signaling pathway to up-regulate inflammatory response and inhibit angiogenesis.

## INTRODUCTION

Although there is no uniform standard, recurrent implantation failure (RIF) most often refers to a failure to achieve a clinical pregnancy in a woman under 40 years of age after transferring at least four high-quality embryos within at least three fresh or frozen cycles, a definition proposed by Coughlan in 2014 [1]. RIF has received increasing attention in the clinical application of *in vitro* fertilization and embryo transfer (IVF-ET), as approximately 15% of women receiving IVF-ET have experienced RIF [2]. Early diagnosis of patients with a high likelihood of RIF facilitates early treatment, may avoid multiple implant failures, and reduce psychological pain and financial losses. RIF is known to involve many factors, including embryo quality, uterine anatomy and endometritis, endocrine and hormone metabolism, maternal immunity, and hematologic factors [3–5]. Among them, the maternal immune system plays a crucial role in the process of embryo attachment and trophoblast invasion. In addition, dysregulation of local endometrial immunity may partially explain RIF.

Embryo implantation can only occur within the endometrium for a limited period of time, and is known as the “window of implantation” (WOI) [6]. During WOI, the endometrium is prepared to receive semi-allogeneic blastocysts through uterine tissue remodeling and endometrial immune microenvironment transformation [7–10]. Macrophages are associated with invasion of the trophoblast and remodeling of tissues and blood vessels in early pregnancy [11]. During the estrus phase of the estrus cycle, dendritic cells (DCs) gather in the uterine cavity or near small blood vessels in the mouse uterus [12], which indicates the implantation site of the embryo. Depletion of macrophages or dendritic cells can lead to implantation failure [11, 13]. Adaptive immune cells, primarily regulatory T cells (Tregs) [14] and regulatory B cells (Bregs) [15], help to protect semi-allogeneic embryos from maternal immune attack by establishing and maintaining immune tolerance. In addition, cytokines and chemokines, such as interleukin-4 (IL-4), leukemia inhibitory factor (LIF), C-X-C motif chemokine ligand 1 (CXCL1), etc. [16, 17], are partly responsible for endometrial receptivity because they regulate the immune response. All of these emphasize the importance of the endometrial immune microenvironment in the implantation process.

The MUC1 gene encodes type I transmembrane glycoprotein, which is expressed on the apical surface of most simple epithelium, including the mammary gland, female reproductive tract, lung, kidney, stomach, gallbladder and pancreas, as well as some non-epithelial cell types. The human MUC1 gene spans 4 to 7 kb and consists of seven exons that can be alternately spliced to form transcripts ranging from 3.7 to 6.4 kb. The full-length proteins contain three domains: short cytoplasmic and transmembrane domains that are highly conserved between species, and large extracellular domains. The human extracellular domain contains 20-125 tandem repeats, with 20 amino acids rich in serine, threonine, and proline residues. Due to these characteristics, the tandem repeat domain has extensive O-glycosylation potential. Proline residues and glycosylation produce rigid extended structures that span much greater distances than most cell surface proteins (including syndecans and integrins). Moreover, in most simple epithelium, including that of the uterus, mucins are not only abundant but also concentrated on the apical surface. It has been shown that MUC1 plays an important role in the immune microenvironment in RIF. This study explores the effect of MUC1 on RIF, and sufficient efforts are needed to fully elucidate the exact mechanism of RIF to find new biomarkers for the diagnosis and treatment of RIF.

## METHODS

### Immune cell infiltration in RIF and its associations with selected hub genes

The expression profiles of RIF and control samples were analyzed using the ImmuInfiltration package in R to estimate the relative proportion of 22 types of immune cells. The association between infiltrative immune cells and selected hub genes was calculated in RIF tissues with the Cell-type Identification by Estimating Relative Subsets of RNA Transcripts (CIBERSORT) package. According to the expression of immune-related hub genes, gene set enrichment analysis (GSEA) was carried out in RIF tissue to identify the possible molecular mechanisms of specific genes in RIF pathogenesis.

### The diagnostic significance of hub genes in RIF

The dataset GSE111974 was utilized to detect the diagnostic efficiency of hub genes in RIF. A receiver operating characteristic curve (ROC) was performed using the pROC package and the diagnostic efficiency of each hub gene was evaluated via the area under the curve (AUC). The hub genes whose AUCs were >0.75 had diagnostic value and were selected for further validation.

### Cell culture and lentivirus transfection

RAW264.7 and THP-1 cells were purchased from Wuhan Pricella Biotechnology Co., Ltd., and cultured in a 37°C incubator using RPMI-1640 medium supplemented with 10% fetal bovine serum (FBS) in 5% CO_2_. RAW264.7 and THP-1 cells were re-suspended in serum-free 1640 medium and cultured in a serum-free incubator at 37°C and 5% CO_2_ for 2 h on a 12-well plate at 5 × 10^6^/well. After 2 hours, the supernatant was gently blown with an eyedropper to wash the non-adherent cells. Subsequently, 1640 medium containing 15% fetal bovine serum was added to each well, and the medium was changed in half on the 3rd and 5th day, respectively, and continued to be cultured at 37°C and 5% CO_2_ in the incubator until the mature RAW264.7 and THP-1 were harvested on the 7th day. Lentivirus infection was performed on day 3 of the culture. Transfected RAW264.7 and THP-1 cells are divided into NC group and MUC1 OE group, and then THP-1 cells are treated with ROS inhibitor N-Acetylcysteine (NAC, 10 μm) and PI3K inhibitor LY294002 (10 μm). RAW264.7 and THP-1 cells of the four groups are stimulated with LPS (20 ng/ml) for 24 h. The small interfering RNA (siRNA) of MUC1 was synthesized by RiboBio (China). The design targets MUC1 with the following sequence: siMUC1 (CGGGATACCTACCATCCTA). Negative control siRNA (NC) was purchased from RiboBio (siN0000001-1-5). Cells were seeded in six-well plates at 60% to 70% confluence and transiently transfected with siRNA (50 nM) using Lipofectamine RNAiMAX Reagent (Thermo Fisher Scientific, USA), and then the following experiments were performed.

### Preparation of the mouse models

32 BALB/C mice were infused with 0.2 mL hydroxyurea and 12 μL epinephrine subcutaneously for 1 week. RAW264.7 cells from NC group, MUC1 OE group, NC group and MUC1 KD group are injected into mice through rat tail vein, with 8 mice in each group. Mice in normal group were infused with 0.2 mL ultra-pure water and 12 μL normal saline subcutaneously for 1 week. After 1 week, the female rats in the normal group were fed with BALB/C males at a ratio of 2:1, and the female rats in the other groups were fed with DBA/2 males at a ratio of 2:1 for 2 days. When vaginal suppositories or vaginal secretions were detected in the morning of the next day, the female rats were recorded as pregnant for 1 day. This study has been approved by the Laboratory Animal Ethics Committee of Hebei North University.

### Immunohistochemical staining

After conventional deaffinity and rehydration, the high-pressure antigen was extracted with sodium citrate solution at pH 6.0 for 3 min, and the sections were incubated at 30°C for 30 min. After blocking endogenous peroxidase with hydrogen peroxide for 30 min, incubated with sections of primary antibody VEGF (Abcam, ab32152, 1:250) overnight at 4°C. The next day, sections were washed and incubated with horseradish peroxidase-conjugated secondary antibody (Abcam, ab97080, 1:200) for 30 minutes. After PBS washing, sections are stained with DAB, hematoxylin staining, dehydrated, gel fixation.

### Flow cytometry

The macrophages in the endometrium of mice were inoculated into 12-well plates with 1 × 10^5^ cells per well. The cells were treated with cytotoxic stimulants or the adherent cell death was triggered by ultraviolet radiation, and the cells were observed with bright field microscopy. 1 × 10^5^ cells were resuspended in 200 μL Binding Buffer with CD86 and CD206 added. Incubation at room temperature for 15 min in a dark place. Fluorescence detection was performed by flow cytometry.

### Western blotting

The THP-1 was inoculated in a 6-well plate and lysis using RIPA (Invitrogen, Carlsbad, CA, USA) for cell lysis and protein extraction. Protein concentrations were detected using the Bicinchoninic Acid (BCA) method (Beyotime, Shanghai, China). We configured 10% sodium dodecyl sulfate - polyacrylamide gel electrophoresis (SDS-PAGE) gel according to the instructions. The protein (30μg) was then added to each well of the SDS-PAGE electrophoresis gel. We then transferred the protein bands onto a polyvinylidene difluoride (PVDF) membrane (Roche, Basel, Switzerland). After washing PVDF membrane with phosphate buffered saline (PBST), we sealed it with 5% skim milk and MUC1, P22, P47, gp91, p-SHP2, p-TBK1, IFN-γ, IL-1β, p-PI3K, p-mTOR, HIF1α, VEGF and GAPDH were incubated with monoclonal diluent solution overnight at 4°C. We then incubated PVDF membranes at room temperature with secondary antibody dilutions for 2 hours. Finally, the protein bands were detected by Electochemiluminescence (ECL).

### Angiogenesis assay

THP-1 cells and HUVEC cells in each group were co-cultured, and the basic medium without FBS was starved for 8 h when the cells reached more than 80% confluent. Melting Matrigel (BD: 356234) at 4°C, spreading Matrigel into the pre-cooled 24-well plate in a super-clean bench with a pre-cooled tip to make the matrix glue evenly distributed, solidifying at 37°C for 30 min. HUVEC cells were digested with trypsin and the cell density was adjusted to 5 × 10^5^ cells/100 μl in serum-free medium. The 100 μl cell suspension was added to the 24-well plate, observed and photographed under an inverted microscope within 4 hours, and the total number of branches, total tube length and total number of rings were observed for each image.

### Statistical analysis

Statistical analysis was performed using SPSS 16.0 software (IBM, Chicago, IL, USA). The level of gene expression was compared using student *t*-test. *P* < 0.05 was considered statistically significant.

## RESULTS

### Immune cell infiltration in RIF

**Figure 1:**
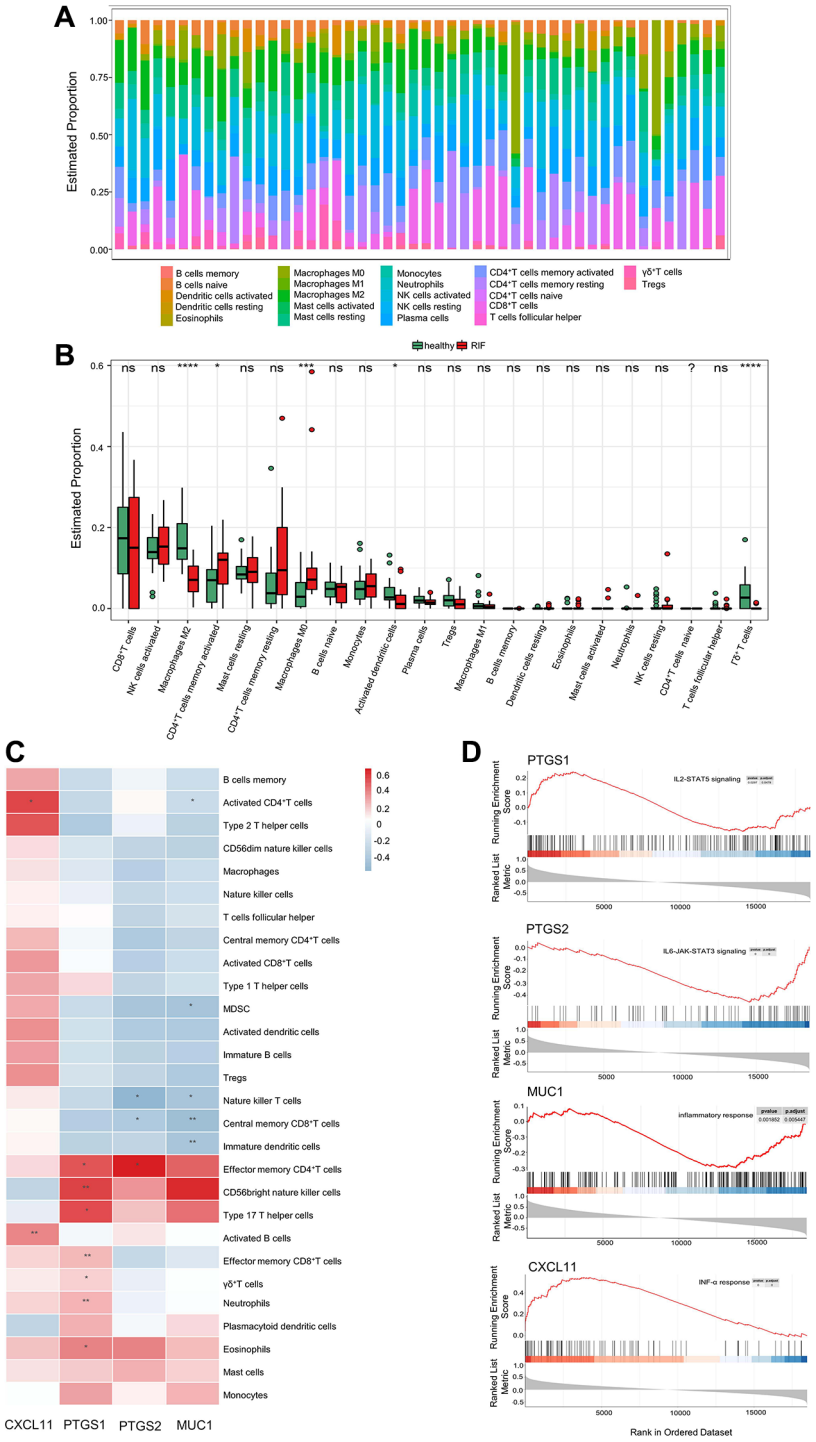
**Endometrial immune characteristics of RIF patients.** (**A**) The landscape of infiltrating immune cells in endometrial tissues of RIF patients and fertile controls. (**B**) The proportion of 22 types of immune cells between RIF patients and fertile controls. (**C**) The associations of selected hub genes and infiltrating immune cells. (**D**) GSEA of selected hub genes in RIF samples. ^*^P < 0.05, ^**^P < 0.01, ^***^P < 0.001, ^***^P < 0.0001, Abbreviations: ns: no significance; RIF: recurrent implantation failure; GSEA: gene set enrichment analysis.

To identify the endometrial immune characteristics of RIF and control samples, the landscape of immune cell infiltration of each sample in GSE111974 was described (Figure 1A). The boxplot compared the relative proportion of 22 types of immune cells between 2 groups. The infiltration levels of activated CD4+ memory T cells and M0 Macrophages significantly increased in RIF samples, whereas M2 Macrophages, activated DCs, and γδT cells markedly declined (Figure 1B). The distribution of the other immune cells in 2 groups was not measurably different.

### The associations between selected hub genes and infiltrated immune cells

To explore the role of selected hub genes in the endometrial immune microenvironment of RIF patients, the associations between selected hub genes and infiltrated immune cells were analyzed using the ImmuInfiltration package (Figure 1C). MUC1 expression was negatively associated with the infiltration of activated CD4+ T cells, CD8+ central memory (CM) T cells, immature DCs, natural killer T cells (NKT cells), and myeloid-derived suppressor cells (MDSC). Similar to MUC1, the level of PTGS2 was inversely related to CD8+ CM T cells and NKT cell infiltration, but positively correlated to CD4+ effector memory (EM) T cell infiltration. PTGS1 expression was positively correlated with the infiltration of effector T cells, helper T cells, neutrophils, eosinophils, and CD56 bright NKT cells. CXCL11 expression was positively associated with the infiltration of activated CD4+ T cell and activated B cell. GSEA results showed that MUC1, CXCL11, PTGS1 and PTGS2 were involved in chemokine signaling, inflammatory response, interferon (INF)-γ response, and tumor necrosis factor (TNF)-α response pathways (Figure 1D); indicating that hub genes may be involved in the immunity and inflammation mechanisms of RIF pathogenesis.

### Diagnostic efficiency of hub genes

**Figure 2:**
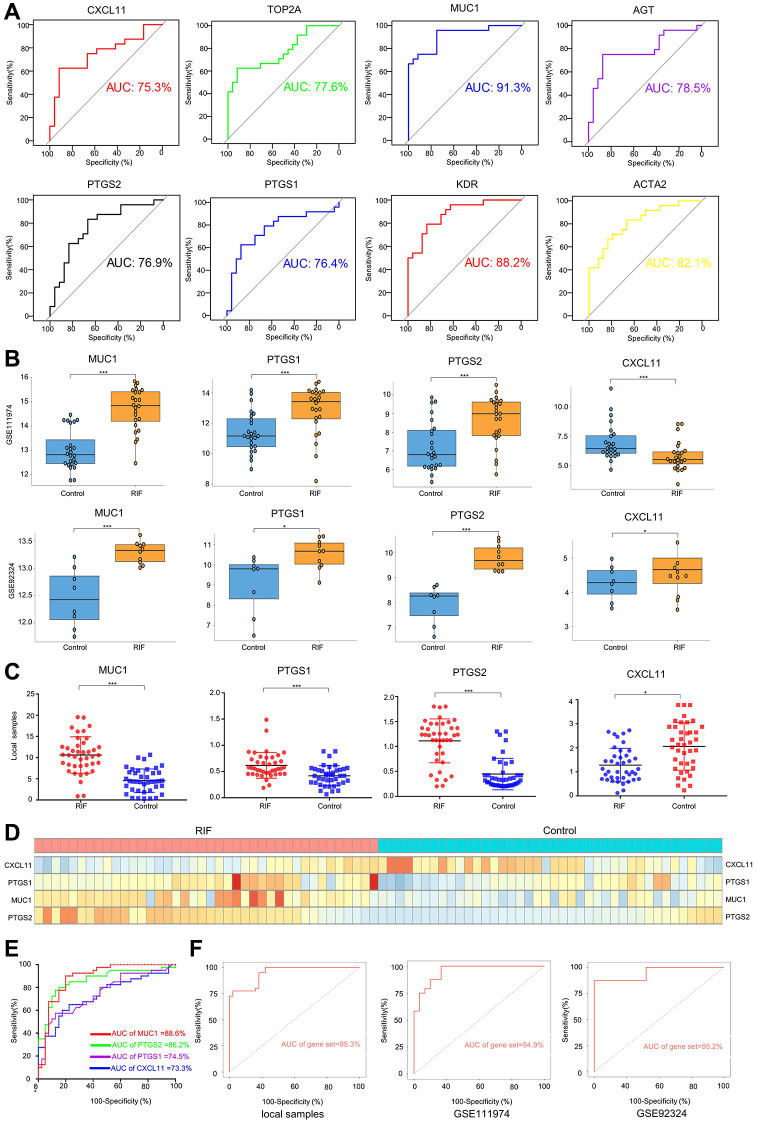
**Diagnostic efficiency of hub genes and validation.** (**A**) ROC analysis of each hub gene using dataset 111974. Only the hub genes whose AUC was higher than 0.75 were selected. The expression levels of selected hub in dataset 111974 (**B**), dataset 92324 (**B**), and local plasma samples (**C**, **D**). (**E**) ROC analysis of each hub gene using local plasma samples. (**F**) ROC analysis of gene set using local plasma samples, dataset111974 and dateset92324. Gene set means a 4-gene (MUC1-PTGS1-PTGS2-CXCL11) signature. ^*^p < 0.05, ^***^p < 0.001. Abbreviations: ROC: receiver operating characteristic; AUC: area under the curve.

To evaluate the diagnostic efficiency of hub genes in RIF, a ROC curve was conducted to analyze the sensitivity and specificity of each hub gene for RIF diagnosis. Our findings showed that MUC1 has the best diagnostic value for distinguishing RIF patients from fertile controls (AUC = 91.3%) (Figure 2A). The hub genes of which the AUC was higher than 0.75 were further verified by another dataset (GSE92324) and 40 local pairs of RIF and control samples. The results revealed that only the expression levels of MUC1, PTGS1, and PTGS2 were stably elevated in endometrial tissue samples of RIF patients, whereas CXCL11 was stably down-regulated in the RIF group (Figure 2B–2D). ROC analysis of local samples demonstrated the promising predictive value of MUC1 (AUC = 88.6%), PGTS2 (AUC = 86.2%), PGTS1 (AUC = 74.5%), and CXCL11 (AUC = 73.3%) for RIF (Figure 2E). A 4-gene signature (MUC1-CXCL11-PTGS1-PTGS2) improved the diagnostic capability of a single gene in local samples (AUC = 95.3%), which was verified in the dataset 111974 (AUC = 94.9%) and dataset 92324 (AUC = 95.2%) (Figure 2F).

### MUC1 can promote M1 polarization and inhibit M2 polarization of macrophages

**Figure 3:**
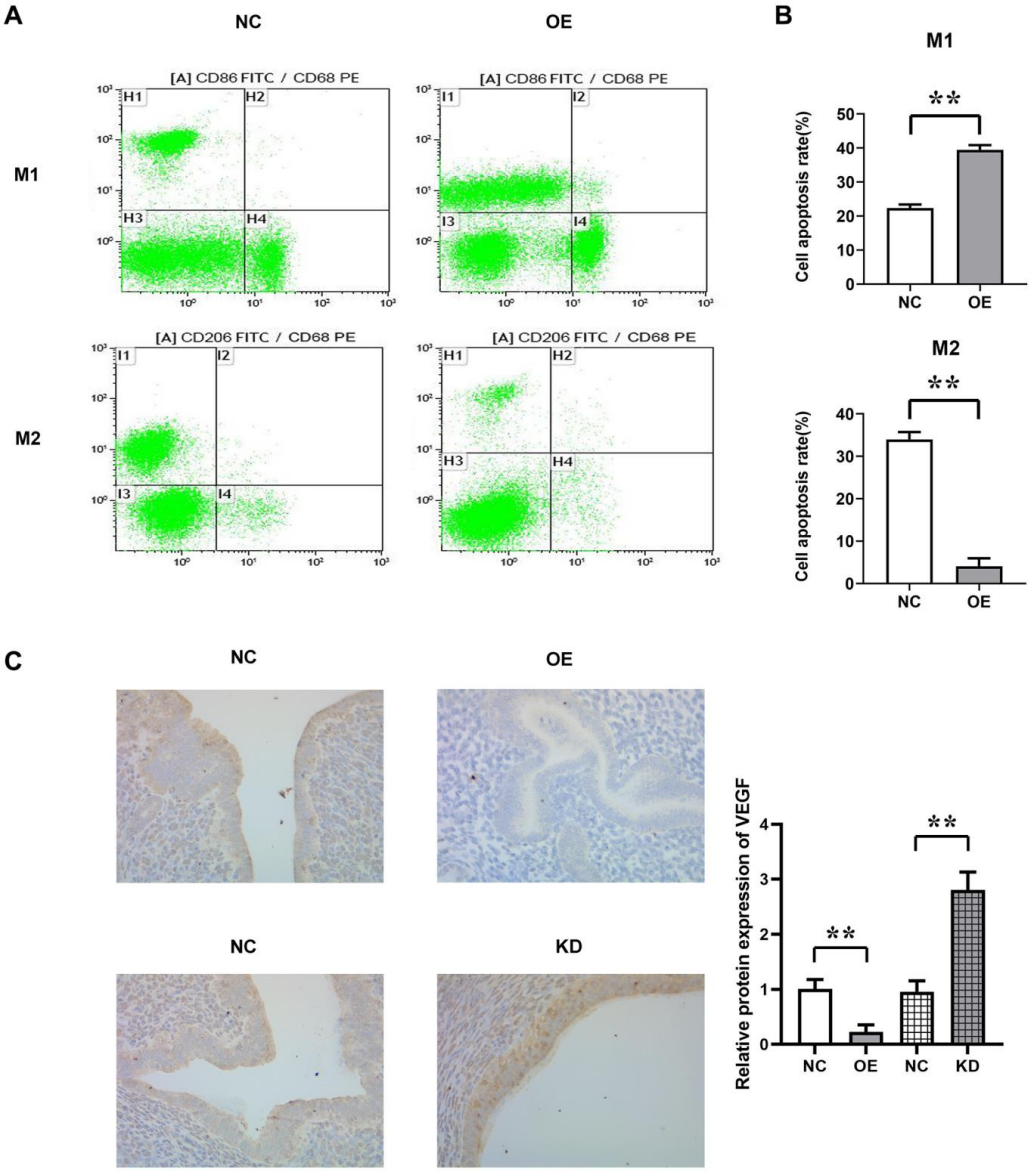
**The proportion of M1 and M2 polarization in macrophages was detected by flow cytometry.** (**A**) Flow cytometry results. (**B**) Statistics of the proportion of M1 and M2 polarization in macrophages; (**C**) Immunohistochemical staining results and relative protein expression statistics of VEGF. *N* = 8; ^**^P < 0.01.

To investigate the effects of MUC1 on macrophages in the endometrial immune microenvironment, we isolated endometrial macrophages from the BABL/C mice with RIF. Then the effect of MUC1 on the polarization of macrophages was detected by flow cytometry. The results showed that compared with the NC group, the proportion of M1 macrophages in MUC1 OE group was significantly increased, while the proportion of M2 macrophages was significantly decreased. The results of immunohistochemical staining showed that the expression of VEGF in the MUC1 OE group was significantly decreased compared with the NC group, while the expression of VEGF in the MUC1 KD group was significantly increased. The results indicated that MUC1 could promote M1 polarization and inhibit M2 polarization of macrophages (Figure 3).

### MUC1 can promote oxidative stress levels and inflammatory responses in macrophages

**Figure 4:**
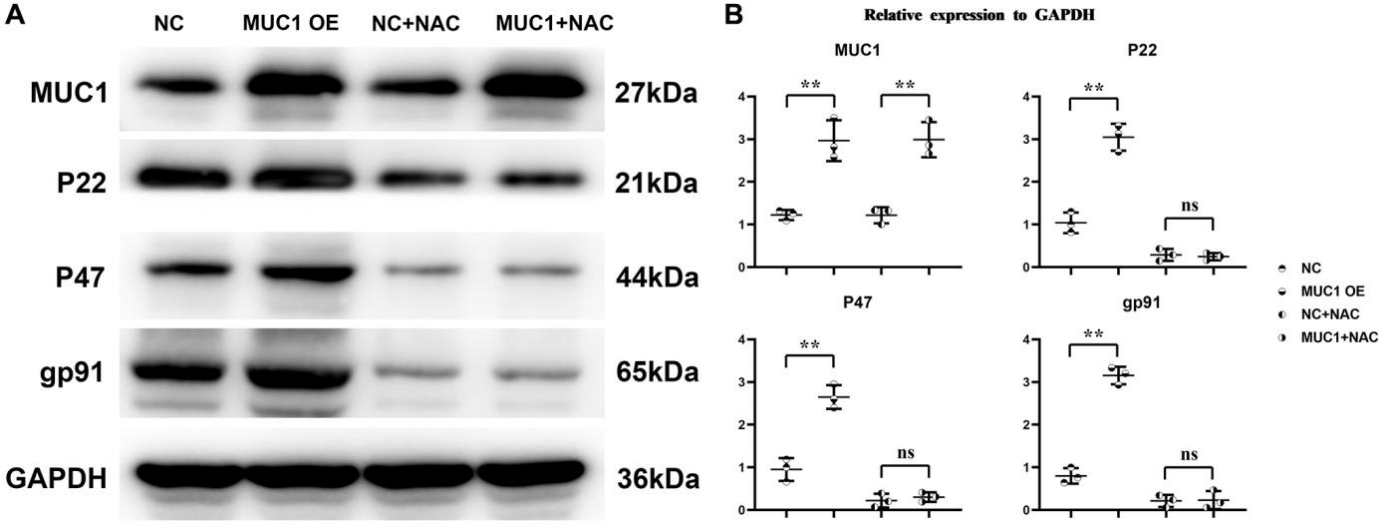
**MUC1 can promote oxidative stress levels in macrophages.** (**A**) Protein bands of MUC1, P22, P47 and gp91. (**B**) Statistics of relative protein expression levels of MUC1, P22, P47 and gp91. *N* = 3; ^**^P < 0.01; ^ns^*P* > 0.05.

**Figure 5:**
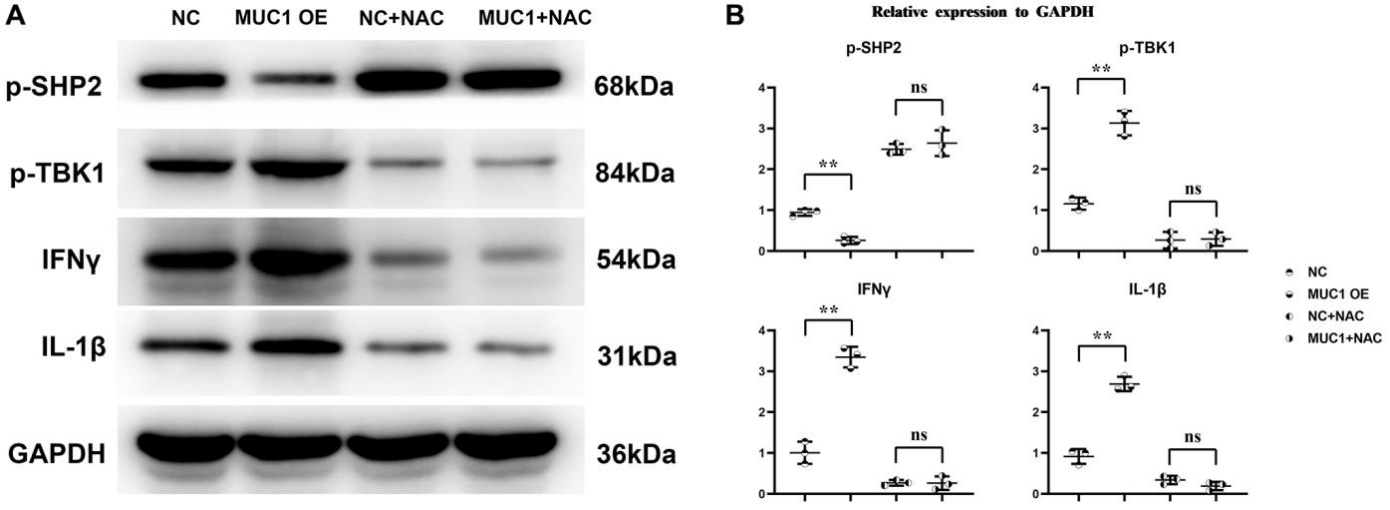
**MUC1 can promote inflammatory responses in macrophages.** (**A**) Protein bands of p-SHP2, p-TBK1, IFN-γ and IL-1β. (**B**) Statistics of relative protein expression levels of p-SHP2, p-TBK1, IFN-γ and IL-1β. *N* = 3; ^**^P < 0.01; ^ns^*P* > 0.05.

To investigate the effect mechanism of MUC1 on RIF. We transfected pLV-MUC1 and negative control into THP-1 cells and THP-1 cells treated them with the ROS inhibitor N-Acetylcysteine (NAC, 10 μm) and PI3K inhibitor LY294002 (10 μm). And then western blotting was used to detect the expression of proteins associated with oxidative stress and inflammatory response. The results showed that compared with the NC group, the relative expression levels of MUC1, P22, P47, gp91, p-TBK1, IFN-γ and IL-1β in MUC1 OE group were significantly increased, while the relative expression levels of p-SHP2 were significantly decreased (Figures 4 and 5). However, after the addition of NAC, the significant differences of P22, P47, gp91, p-TBK1, IFN-γ and IL-1β between the NC group and the MUC1 OE group were eliminated. The results suggest that MUC1 can promote oxidative stress and inflammation through ROS-SHP2 signaling pathway in macrophages.

### MUC1 can inhibit angiogenesis

**Figure 6:**
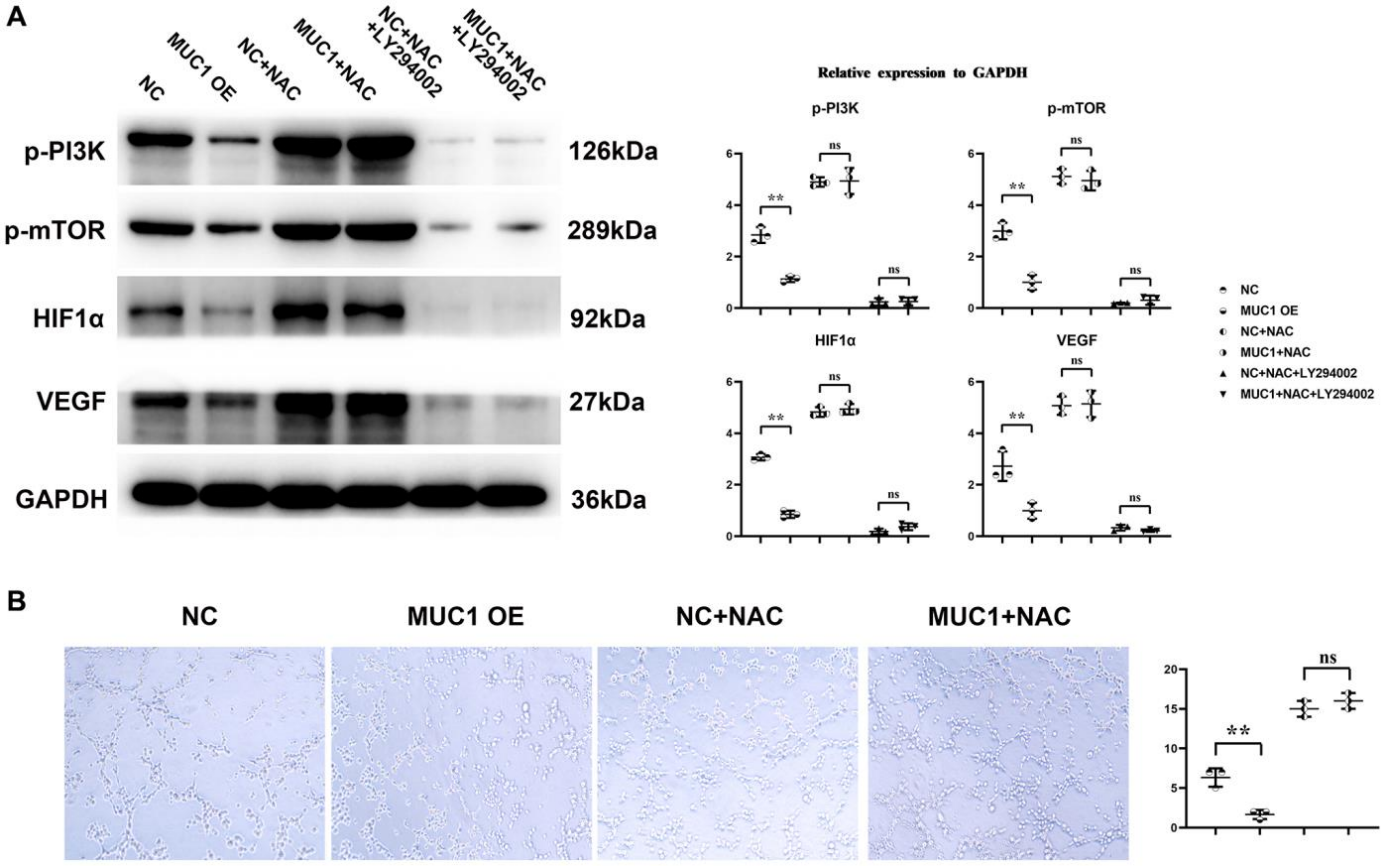
**MUC1 can inhibit angiogenesis.** (**A**) Statistics of protein bands and relative protein expression levels of p-PI3K, p-mTOR, HIF1α and VEGF. (**B**) Diagram of experimental results of angiogenesis and statistics of the number of angiogenesis. *N* = 3; ^**^P < 0.01; ^ns^*P* > 0.05.

**Figure 7:**
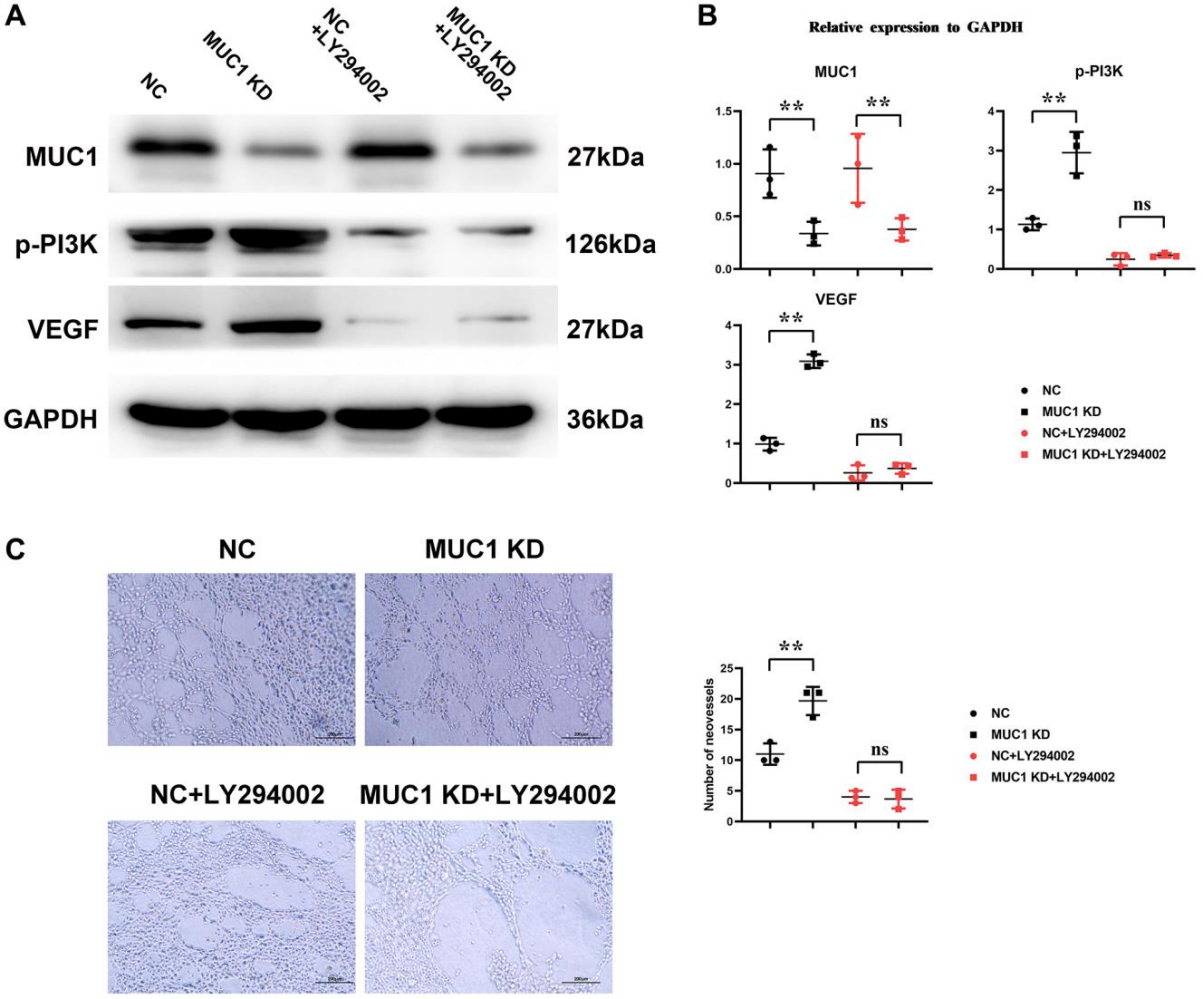
**Inhibition of MUC1 can promote angiogenesis.** (**A**) Protein bands of MUC1, p-PI3K and VEGF. (**B**) Statistics of relative protein expression levels of MUC1, p-PI3K and VEGF. (**C**) Diagram of experimental results of angiogenesis and statistics of the number of angiogenesis. *N* = 3; ^**^P < 0.01; ^ns^*P* > 0.05.

**Figure 8:**
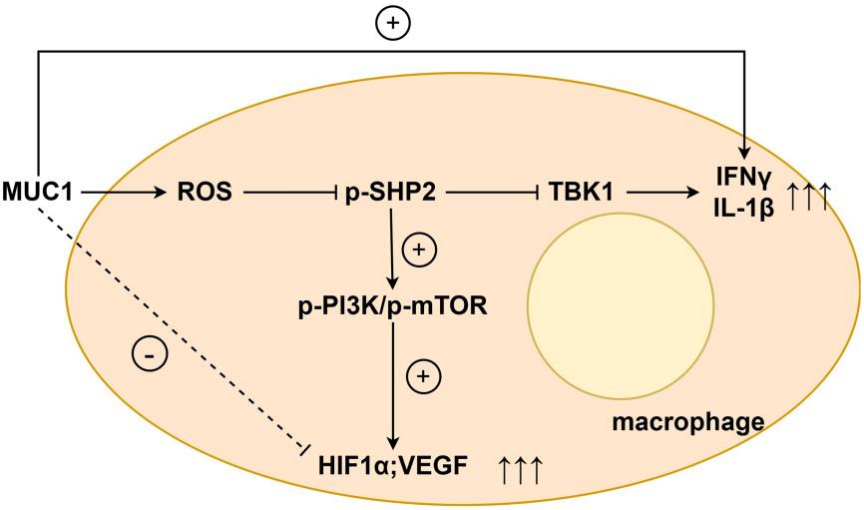
MUC1 promotes RIF by regulating macrophage ROS-SHP2 signaling pathway to up-regulate inflammatory response and inhibit angiogenesis.

Western blotting results showed that compared with the NC group, the expressions of p-PI3K, p-mTOR, HIF1α and VEGF in the MUC1 OE group were significantly reduced. However, the relative protein expressions of p-PI3K, p-mTOR, HIF1α and VEGF between the NC group and the MUC1 OE group were increased after the addition of NAC, and the significant differences were eliminated. After the addition of LY294002, the relative protein expressions of p-PI3K, p-mTOR, HIF1α and VEGF between the NC group and the MUC1 OE group were significantly reduced, and there was still no significant difference between the two groups. The results of angiogenesis assay showed that compared with the NC group, the number of angiogenesis was significantly reduced in the MUC1 OE group. However, after the addition of NAC, the number of blood vessels between the NC group and the MUC1 OE group was significantly increased, and the significant difference was eliminated. The number of blood vessels was significantly reduced between the NC group and the MUC1 OE group after the LY294002 was added, and there was still no significant difference between the two groups (Figure 6). These results suggest that MUC1 inhibits angiogenesis by regulating macrophage ROS- SHP2 signaling pathway. We then designed the MUC1 KD group as well as a negative control group to continue to validate the above conclusions. The results of Western blotting showed that the relative protein expression of MUC1 in the MUC1 KD group was significantly lower than that of the NC group, and the relative protein expression of p-PI3K and VEGF was significantly increased. However, after the addition of LY294002, the significant differences between the relative protein expressions of MUC1, p-PI3K, and VEGF in the NC group and the MUC1 KD group were eliminated. The results of cathetogenic experiments showed that the number of blood vessels in the MUC1 KD group was significantly higher than that in the NC group. However, after the addition of LY294002, the significant difference in the number of blood vessels between the NC group and the MUC1 KD group was eliminated (Figure 7). Therefore, it can be concluded that MUC1 promotes RIF by regulating the macrophage ROS-SHP2 signaling pathway, thereby upregulating the inflammatory response and inhibiting angiogenesis (Figure 8).

## DISCUSSION

RIF has been one of the major problems in assisted reproduction research, and it brings financial and psychological stress to patients. Studies have shown that some of the major immune cell types that identify RIF patients, including NK cells, CD4 T cells, CD8 T cells, Treg, macrophages, three groups of innate lymphocytes, B cells, circulating lymphocytes, and B cell dilatation levels were lower in the tregRIF group than in the control group [18]. Immunotherapy is being considered as a potential intervention to treat RIF. Peripheral blood mononuclear cell (PBMC) therapy and intravenous immunoglobulin have been reported to improve pregnancy outcomes in women with RIF. In addition, identifying DEGs in the endometrium of RIF is an important way to elucidate the pathogenesis of RIF. Therefore, this study provides a potential biomarker for the diagnosis and treatment of RIF by exploring the dysregulation gene MUC1 associated with RIF, and exploring the molecular mechanism of the inhibitory effect of MUC1 on macrophages in the immune microenvironment of RIF.

The hub gene and the matrix of infiltrating immune cells showed that MUC1, CXCL11, PTGS1 and PTGS2 were associated with different immune cells. The GSEA results of MUC1, CXCL11, PTGS1 and PTGS2 genes showed that the pathway involved in each hub gene was not the same, which further indicated that each hub gene had its own value and could not be replaced by another hub gene. GSE111974 dataset was used for ROC analysis, and it was found that the expressions of MUC1, PTGS2, PTGS1 and CXCL11 had good diagnostic value, and AUC was higher than 0.75. After validation of the GESE92324 dataset and local samples, MUC1, CXCL11, PTGS1 and PTGS2 were labeled as potential biomarkers for the diagnosis of RIF, and the four-gene signature (MUC1-CXCL11-pTGs1-PTGS2) showed perfect discrimination ability with AUC of up to 95%.

MUC1, located in the endometrial lumen epithelium, is a glycoprotein with a short cytoplasmic domain and a large extracellular tail that is a key component of the innate immune system. It can act as a protective barrier against microbial and proteolytic attacks [19]. *In vivo* experimental results showed that MUC1 could promote M1 polarization and inhibit M2 polarization of macrophages in RIF immune microenvironment. The results of immunohistochemical staining showed that MUC1 could inhibit the expression of VEGF. Studies have shown that MUC1 can increase the level of oxidative stress. Western blotting results showed that MUC1 overexpression promoted the relative protein expression of P22, P47 and gp91 in macrophages. Moreover, NADPH oxidase mediated intracellular ROS production, thereby inducing the oxidative inactivation of SHP-2. SHP2 can restrict the expression of NLRP3 by inhibiting ANT1 and mitochondrial dysfunction [20]. The C-terminal domain of SHP-2 directly binds to TANK-binding kinase (TBK1) by interacting with the kinase domain of TBK1. SHP-2 deficiency increases TBK1 activation and IFN-γ expression. Therefore, we found by Western blotting that MUC1 overexpression can promote the relative protein expression of p-TBK1, IFN-γ and IL-1β in macrophages, and inhibit the relative protein expression of p-SHP2. It has been found that the activation of SHP-2 can promote the activation of PI3K/mTOR signaling pathway, which can promote the relative protein expression of cell growth factor HIF1α and VEGF [21–23]. Therefore, we verified by Western blotting and angiogenesis experiments, and found that MUC1 overexpression could inhibit the activation of PI3K/mTOR signaling pathway, thereby inhibiting the expression of HIF1α and VEGF and the amount of angiogenesis. However, after the addition of ROS inhibitors, we found that significant differences between the NC group and the MUC1 OE group were eliminated, except for the expression of MUC1. Western blotting results showed that inhibition of MUC1 could increase the relative protein expression of p-PI3K and VEGF and the number of angiogenesis, while LY294002 would eliminate the effect of MUC1 inhibition.

In summary, we analyzed the characteristics of the endometrial immune microenvironment of RIF and identified a four-gene signature (MUC1-CXCL11-PTGS1-PTGS2) with diagnostic value. Moreover, we found that MUC1 promotes RIF by regulating macrophage ROS-SHP2 signaling pathway, thereby up-regulating inflammatory response and inhibiting angiogenesis. The results of this study will contribute to the early diagnosis of RIF and a deeper understanding of the endometrial immune microenvironment characteristics of RIF, which will contribute to early diagnosis and therapeutic intervention.
